# Patient-Reported Outcomes after Surgical, Endoscopic, or Radiological Techniques for Nutritional Support in Esophageal Cancer Patients: A Systematic Review

**DOI:** 10.3390/curroncol31100460

**Published:** 2024-10-14

**Authors:** Filipa Fontes, Davide Fernandes, Ana Almeida, Inês Sá, Mário Dinis-Ribeiro

**Affiliations:** 1 Precancerous Lesions and Early Cancer Management Group IPO Porto Research Center (CI-IPOP), Portuguese Oncology Institute of Porto (IPO Porto)/Porto Comprehensive Cancer Centre (Porto.CCC) & RISE@CI-IPOP (Health Research Network), Rua Doutor António Bernardino de Almeida, 4200-072 Porto, Portugal; mario.ribeiro@ipoporto.min-saude.pt; 2 Public Health and Forensic Sciences, and Medical Education Department, Faculty of Medicine, University of Porto, Rua Doutor Plácido da Costa, 4200-450 Porto, Portugal; 3 Department of Imaging Sciences and Radioncology, Portuguese Oncology Institute of Porto, Rua Doutor António Bernardino de Almeida, 4200-072 Porto, Portugal; davide.miguel@ipoporto.min-saude.pt (D.F.); ana.m.almeida@ipoporto.min-saude.pt (A.A.); 4 Oncology Nursing Research Unit IPO Porto Research Center (CI-IPOP), Portuguese Oncology Institute of Porto (IPO Porto)/Porto Comprehensive Cancer Centre (Porto.CCC) & RISE@CI-IPOP (Health Research Network), Rua Doutor António Bernardino de Almeida, 4200-072 Porto, Portugal; 5 Department of Gastroenterology, Portuguese Oncology Institute of Porto, Rua Doutor António Bernardino de Almeida, 4200-072 Porto, Portugal; ines.andre@ipoporto.min-saude.pt

**Keywords:** esophageal neoplasms, gastrostomy, jejunostomy, patient-reported outcomes, self-expandable metallic stents

## Abstract

Several techniques exist to maintain oral and/or enteral feeding among esophageal cancer (EC) patients, but their impact on patient-reported outcomes (PROs) remains unclear. This systematic review aimed to assess the impact of nutritional support techniques on PROs in EC patients. We searched Medline, Web of Science, and CINAHL Complete from inception to 3 April 2024. Eligible studies included those evaluating EC patients, reporting PROs using standardized measures, and providing data on different nutritional support techniques or comparing them to no intervention. The reference lists of the included studies were also screened for additional eligible articles. The Mixed Methods Appraisal Tool was used to evaluate the quality of the included studies. Of the 694 articles identified from databases and 224 from backward citation, 11 studies met the inclusion criteria. Nine studies evaluated the overall quality of life (QoL), four assessed pain, and one evaluated depression. Among those submitted to esophagectomy, jejunostomy may be associated with higher QoL scores and less postoperative pain, compared to a nasojejunal tube, but no significant differences were found when compared to no intervention. For patients undergoing chemotherapy or receiving palliative/symptomatic treatment, expandable metal stents (SEMSs) were associated with higher levels of emotional functioning when compared with laparoscopic gastrostomy. Moreover, percutaneous endoscopic gastrostomy or SEMSs were associated with a higher QoL compared with nasogastric tubes. This review underscores the importance of considering PRO measures when evaluating nutritional support techniques in cancer patients, though further robust evidence is needed to fully understand these associations.

## 1. Introduction

Worldwide, esophageal cancer ranks as the eleventh most frequent cause of cancer and stands as the seventh leading cause of cancer-related mortality, with approximately 510,000 new cases and 445,000 deaths reported in 2022 [1]. Despite advances in diagnosis and treatment, the prognosis of esophageal cancer remains poor in most countries, with a 5-year age-standardized net survival ranging from 5.6% to 23.9%, among individuals diagnosed between 2010 and 2014 in Europe [2].

Malnutrition and its associated complications, including weight loss and sarcopenia, are extremely prevalent among esophageal cancer patients [3,4,5]. This is often attributed to physical obstruction by the tumor, in cases of advanced stages of the disease, leading to dysphagia, as well as the adverse effects of cancer treatments, which may impede nutritional intake due to swallowing difficulties, mucositis, and anorexia [6,7]. Moreover, metabolic alterations induced by the cancer-associated systemic inflammatory response may also contribute to these nutritional challenges [6,7]. In response to these challenges, the European Society for Clinical Nutrition and Metabolism (ESPEN) recommends enteral nutrition in the presence or anticipation of the inability to eat adequately (e.g., less than 50% of the recommended for more than 7 days or only 50–75% of the requirement for more than 14 days) [8,9]. In addition, ESPEN stated that whenever feasible, the oral/enteral route shall be preferred [9].

Several techniques, including surgical, endoscopy, or radiological, are available to allow the maintenance of oral and/or enteral feeding, depending on the indication (e.g., short-term or long-term enteral nutrition or palliation of dysphagia) [10,11,12]. For instance, percutaneous endoscopic gastrostomy (PEG) is widely used for long-term enteral nutrition, while radiologically inserted gastrostomy (RIG) is used as an alternative for patients with significant comorbidities and a high anesthesia risk. Surgical jejunostomy is often chosen for patients performing surgical resection requiring postoperative enteral feeding, but both nasogastric tube (NGT) feeding and nasojejunal tube (NJT) feeding are alternatives. Esophageal stents are recommended for palliative patients with dysphagia or malignant fistula; however, their use should be postponed in patients undergoing radiotherapy, considering alternative options [10,12]. Although the advantages and disadvantages of each technique, particularly in terms of complication rates, are well described [10,11,12,13], the impact of these techniques on patient-reported outcomes (PROs), such as quality of life (QoL), remains poorly understood. Therefore, we aimed to systematically evaluate the effect of nutritional support techniques—such as jejunostomy, NJT, NGT, and stents, on PROs, with a specific focus on determining which technique most significantly improves the QoL in esophageal cancer patients.

## 2. Materials and Methods

This systematic review was conducted according to the Preferred Reporting Items for Systematic Reviews and Meta-Analyses (PRISMA) guidelines [14].

### 2.1. Search Strategy

We searched Medline (using PubMed), Web of Science, and CINAHL Complete (via EBSCOhost) from inception to 3 April 2024 with the following search expression: (“esophageal cancer” OR “oesophageal cancer” OR “esophageal neoplasm” OR “oesophageal neoplasm”) AND (gastrostomy OR jejunostomy OR “esophageal stent” OR “oesophageal stent” OR “enteric stent” OR gastrojejunostomy OR “nasojejun*” OR nasogastric) AND (self-report* OR patient-reported* OR preference* OR experience* OR perception* OR measure* OR depress* OR anxiety OR symptom* OR sleep* OR “quality of life” OR “health status” OR pain).

### 2.2. Eligibility Criteria

We included observational, quasi-experimental, and experimental studies that assessed the impact of surgical, endoscopic, or radiological techniques for providing nutritional support to esophageal cancer patients on PROs. The exclusion criteria were as follows: (i) studies not involving esophageal cancer patients or which did not allow the retrieval of data for only those with esophageal cancer; (ii) case reports, case series, qualitative research studies, guidelines, non-systematic reviews, or systematic reviews not addressing the effect of surgical, endoscopic, or radiological techniques for the nutritional support of esophageal cancer patients on PROs; (iii) studies lacking data for comparing PROs among different nutritional support techniques (or comparing any technique with no specific intervention); and (iv) studies lacking data on PROs evaluated with standardized measures (e.g., validated questionnaires or scales). In addition, we excluded studies comparing different materials of the same technique (for instance, different types of esophageal stents). There were no language or temporal restrictions.

For the purpose of the present review, a PRO was considered any report of the status of a patient’s health condition that comes directly from the patient without the interpretation of the patient’s response by a clinician or anyone else [15].

### 2.3. Study Selection

The selection of papers for this review was independently conducted by two researchers (D.F. and A.A), according to predefined criteria, utilizing Covidence systematic review management software [16]. Discrepancies in their assessments were resolved through discussion or by a third reviewer (F.F.). After importing references into Covidence and removing duplicate articles, we initially screened the reference list based on titles and abstracts. Subsequently, we conducted a detailed assessment of those not excluded in the initial screening. The reference lists of the studies selected for inclusion in the systematic review were also screened using the same criteria to identify additional eligible reports.

### 2.4. Data Extraction

From each paper included in the systematic review, we collected data concerning the country where the study was conducted, the study design, the aim of the study, the study population, the sample size, the sample characteristics (age and cancer stage), the technique and the PRO evaluated in each study, the timing of assessment, the estimates of the association between the technique for nutritional support and PROs (or the necessary information to compute them), and strategies used to control confounding, whenever applicable. For three papers [17,18,19], data were extracted from figures using WebPlotDigitizer [20]. When studies reported evaluations at different time points after baseline, the results corresponding to the longest follow-up were extracted. In cases where studies used multiple tools to assess the same PRO (for instance, both the core Quality of Life Questionnaire (QLQ-C30) of the European Organization for Research and Treatment of Cancer (EORTC) and an esophageal-cancer-specific module), data were extracted from the most used questionnaire among the included studies to allow comparison between studies. Data extraction was independently conducted by two researchers (D.F. and A.A.), using a customized form; any discrepancies were resolved with the involvement of a third researcher (F.F.).

### 2.5. Quality Assessment

The methodological quality evaluation of included studies was independently assessed by two reviewers, using the Mixed Methods Appraisal Tool (MMAT), version 2018. This tool was designed to assess the quality of different study designs [21]. The authors of the MMAT discourage the calculation of an overall score from the ratings of each criterion. Therefore, we presented a detailed consensus rating from the two reviewers for each criterion. For the purposes of result synthesis and discussion, we considered studies achieving fewer than three MMAT criteria as low-quality, those achieving three as medium-quality, and those achieving at least four out of the five criteria as high-quality.

### 2.6. Data Synthesis

Due to the heterogeneity of the study populations, the techniques compared, and the options used by the authors for summarizing the results, it was not possible to perform a quantitative synthesis of the results. Therefore, the impact of the different techniques used on PROs was analyzed considering the direction and the statistical significance of the associations observed when compared each technique with a reference group (other technique or no specific technique). Whenever necessary and where data were available, additional comparisons between techniques were computed, using the *t*-test statistics. Results were considered statistically significant for *p*-values less than 0.05.

As most studies evaluate the impact of surgical, endoscopy, or radiological techniques for nutritional support on the QoL at a single point in time (instead of the variation between periods, i.e., before and after the procedure) using the QLQ-C30 from the EORTC, the summarized results of these studies are presented using figures. However, all results, regardless of the method used to evaluate the PROs and the specific PROs evaluated, are summarized in the text.

## 3. Results

**Figure 1:**
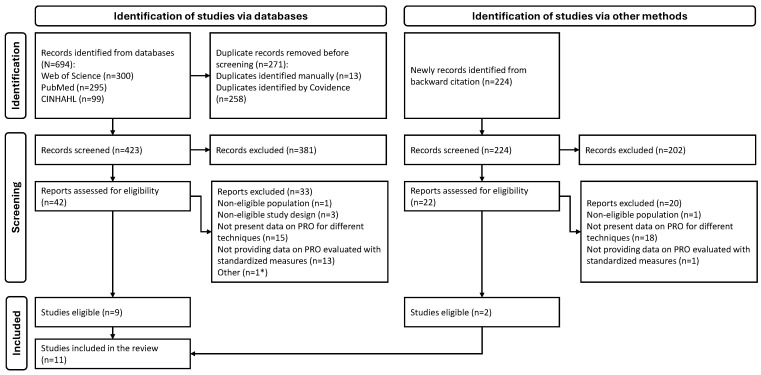
Systematic review flowchart. PRO, patient-reported outcome. * One article written in Chinese was excluded because an accurate translation was not possible.

A detailed flowchart describing the study selection process is presented in Figure 1. From a total of 694 studies identified from databases, and from the additional 224 articles identified through backward citation, a total of 11 studies met the inclusion criteria and were included in this systematic review [17,18,19,22,23,24,25,26,27,28,29], which are described in detail in Appendix A.

Most of the investigations were conducted in Asia (five studies from China [18,22,23,26,27], one from Taiwan [28], one from the USA [25], one from Sweeden [24], one from Italy [17], one from the Netherlands [19], and one from the United Kingdom [29]). The median sample size of the included studies was 120, ranging from 27 to 766. Among the included studies, four focused exclusively on patients with squamous cell carcinoma [18,22,23,28], while seven included patients with both squamous cell carcinoma and adenocarcinoma [17,19,24,25,26,27,29]. Most studies included patients with cancer stages ranging from 0/I to III/IV [17,18,22,24,26,27,28], while one study included only participants with stage IV cancer [25], and for three studies, information on cancer stage was not available [19,23,29]. Four studies evaluated patients undergoing esophagectomy [17,18,24,27], two during chemoradiotherapy [22,28], and the others during palliative care or the management of dysphagia or tracheoesophageal/bronchoesophageal fistula [19,23,25,26,29].

Table 1 provides a detailed description of the PROs evaluated and the instruments used for their evaluation across the included studies. Nine studies assessed the effect of techniques for nutritional support on the QoL [17,18,19,22,23,24,27,28,29], four studies evaluated pain [19,25,26,27], and one study assessed depression [28]. Concerning studies evaluating the QoL, all studies used the core Quality of Life Questionnaire (QLQ-C30) from the European Organization for Research and Treatment of Cancer (EORTC) [17,18,19,22,23,24,27,28,29], five also reported data using an EORTC esophageal-cancer-specific module (QLQ-OES) [17,19,23,24,28], two employed the Euroqol-5D questionnaire [19,29], and one used the Spitzer Quality of Life Index [29]. For pain evaluation, three studies used the Visual Analog Scale [19,26,27], and one study employed a Likert scale [25]. The depression module of the Patient Health Questionnaire was used in the study assessing depression [28].

The assessment of the methodological quality of the studies is summarized in Table 2. According to the classification proposed by the MMAT, three studies were classified as quantitative randomized controlled trials (RCTs) [18,19,29] and the others as quantitative non-randomized (three prospective cohort studies [22,24,27], three retrospective cohort studies [17,25,26], and two non-randomized controlled trials [23,28] (Appendix A)). In the RCTs, all methodological quality criteria were positively evaluated, except for the blinding of outcome assessors and, in one study, the appropriateness of randomization. Two of these RCTs were classified as high-quality, while one was rated as medium-quality. Among the non-randomized studies, five did not control for the effect of potential confounders [22,23,26,27,28]. Overall, seven non-randomized studies were classified as high-quality and one as medium-quality.

### 3.1. Overall Quality of Life

**Figure 2:**
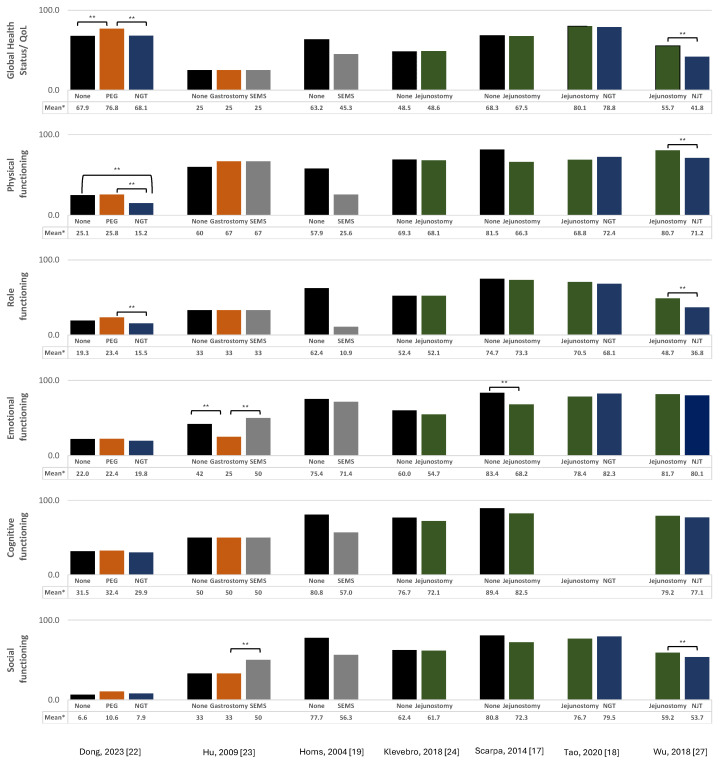
Association between surgical, endoscopic, or radiological techniques for nutritional support of esophageal cancer patients and EORTC QLQ-C30 global health status/ functional scales. * Mean EORTC QLQ-C30 scores are displayed for all studies except for Hu, 2009 [23], which presented medians instead. Higher scores for the global health status and for the functional scales represent a healthy level of quality of life and functioning, respectively; range: 0–100. ** indicates statistically significant differences in the global health status/QoL or in the function scale, as applicable, between the two indicated techniques (*p*-value < 0.05).

**Figure 3:**
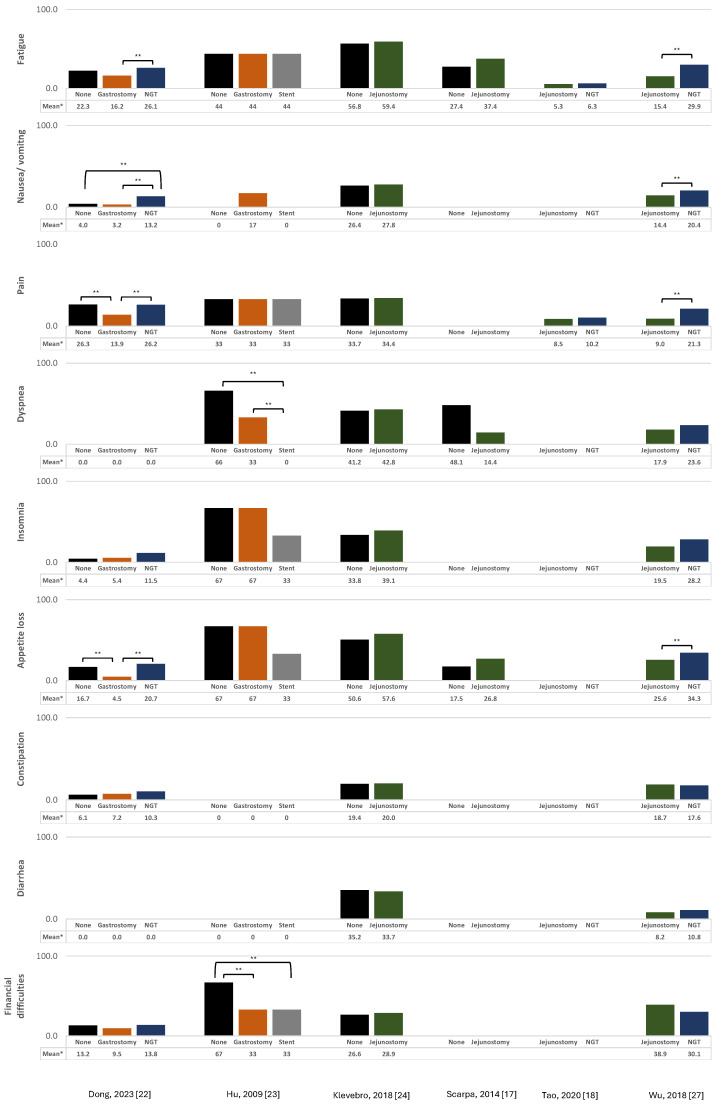
Association between surgical, endoscopic, or radiological techniques for nutritional support of esophageal cancer patients and EORTC QLQ-C30 symptom scales and symptom scales/items. * Mean EORTC QLQ-C30 scores are displayed for all studies except for Hu, 2009 [23], which presented medians instead. Higher scores for the symptom scales/items represent a higher level of symptomatology/problems; range: 0–100. ** indicates statistically significant differences in the global health status/QoL or in the function scale, as applicable, between the two indicated techniques (*p*-value < 0.05).

The results from the studies evaluating the association between surgical, endoscopy, or radiological techniques for the nutritional support of esophageal cancer patients and EORTC QLQ-C30 global health status and functional and symptom scales/items are summarized in Figure 2 and Figure 3.

#### 3.1.1. Jejunostomy

The impact of jejunostomy on the QoL was evaluated in four studies (one RCT [18] and three cohort studies [17,24,27]). Two studies compared jejunostomy with no intervention [17,24], while the others compared jejunostomy with NGTs [18] or NJTs [27]. There were no statistically significant differences observed in the global health status/QoL score or functional/symptom scales when jejunostomy was compared to no intervention [17,24], except for lower emotional functioning in one study [17]. Concerning the comparisons with NGT/NJT, one study demonstrated significantly higher values of global health status and physical, role, and social functioning for those with jejunostomy when compared to NJT [27], while no significant differences were observed in the comparison between jejunostomy and NGT [18]. Also, among the former, there were significantly lower levels of fatigue, nausea/vomiting, pain, and appetite loss in the jejunostomy group [27].

#### 3.1.2. Nasogastric/Nasojejunal Tube

A total of four studies evaluated the impact of NGT or NJT on the QoL (one RCT [18], one non-randomized controlled trial [28], and two cohort studies [22,27]). NGT were associated with significantly lower levels of global health status/QoL, physical functioning, and role functioning, as well as higher levels of fatigue, nausea/vomiting, pain, and appetite loss compared to PEG [22]. Compared to no intervention, patients with an NGT presented lower levels of physical functioning and higher levels of nausea/vomiting [22]. Patients with an NGT had significantly higher levels of aggravation of insomnia over time compared to those with an esophageal self-expandable metal stent (SEMS) [28]. The results for the comparison between NJT or NGT with jejunostomy [18,27] are presented in the previous subchapter.

#### 3.1.3. Gastrostomy

The impact of gastrostomy on the QoL was evaluated in two studies (one non-randomized controlled trial [23] and one cohort study [22]). One compared laparoscopic gastrostomy with no intervention and SEMSs [23], while the other compared PEG with no intervention and NGT [22]. The results from the non-randomized trial indicated that patients with laparoscopic gastrostomy had significant lower emotional and social functioning compared to those with an SEMS but significantly higher levels of dyspnea. [23] Additionally, when compared to no intervention, they presented significantly lower emotional functioning and higher financial difficulties [23]. In the cohort study, PEG was associated with statistically significant higher levels of global health status/QoL, when compared to both NGT and no intervention, as well as higher levels of physical and role functioning when compared to NGT, as previously reported. Furthermore, individuals with PEG reported significantly lower levels of pain and appetite loss, compared to no intervention [22].

#### 3.1.4. Esophageal Stent

The impact of esophageal stents on the QoL was evaluated in four studies (two RCTs [19,29] and two non-randomized controlled trials [23,28]). Two studies compared SEMSs with no specific intervention [19,29], one compared SEMSs with laparoscopic gastrostomy and no intervention [23], and another with NGT and with no intervention [28]. In studies comparing SEMSs with no specific intervention, one study reported no statistically significant differences in any of the EORTC QLQ-C30 scores at 12 months after treatment [19], while another reported a significantly worse QoL, assessed using the Spitzer Quality of Life Index, in patients submitted to an SEMS compared to those without a stent [29]. Another study showed that SEMSs were significantly associated with lower levels of dyspnea and financial difficulties compared to no intervention [23], while other found a worsening of emotional function and insomnia over time [28]. Regarding the comparison with laparoscopic gastrostomy and NGTs [23,28], the results are described in the “Gastrostomy” and “Nasogastric/Nasojejunal Tube” subchapters, respectively.

### 3.2. Pain

Among the four studies evaluating pain (one RCT [19] and three cohort studies [25,26,27]), one study compared SEMSs with RIG [26], two studies compared stent insertion (no type specified) and SEMSs, as applicable, with no specific intervention [19,25], and another study compared jejunostomy with NJTs [27]. In the former study, a significantly higher proportion of patients experienced local severe pain (defined as a score of 7 or higher on the Visual Analog Scale) following SEMS insertion compared to those undergoing RIG (21.3% vs. 0.0%; *p* < 0001) [26]. Regarding comparisons of SEMSs with no intervention, one study reported that patients who did not undergo stent insertion but received palliative radiotherapy experienced more rapid and sustained pain relief over time (*p* < 0.001) [25]. Conversely, in another study, no statistically significant differences in pain scores were observed at 12 months after treatment between patients with SEMSs and those without SEMSs (but who underwent brachytherapy) [19]. In the comparison between jejunostomy and NJTs, patients in the jejunostomy group had significantly less pain in the first 3 days after surgery compared to those in the NJT group [27].

### 3.3. Depression

One non-randomized controlled trial evaluated depression and found that patients with an SEMS exhibited a statistically significant increase in depression levels over time (mean difference between), while those in the no intervention group experienced a decrease over time; those with an NGT showed no significant differences in depression levels over time (*p* < 0.01) [28].

## 4. Discussion

This systematic review provides an overview of the available evidence on the association between different techniques for the nutritional support of esophageal cancer patients and PROs, including the QoL, pain, and depression. Despite the limited and inconsistent results across available studies, it appears that among those submitted to esophagectomy, jejunostomy may be associated with a greater QoL and less postoperative pain compared to NJT. For patients undergoing chemotherapy or receiving palliative/symptomatic treatment, NGT were associated with a lower QoL compared to no intervention, PEG, and SEMSs. Additionally, laparoscopic gastrostomy was associated with lower levels of emotional functioning compared to both SEMSs and no intervention.

### 4.1. Jejunostomy vs. Alternatives

Previous evidence has demonstrated that for those submitted to surgical treatment, a surgical jejunostomy is an effective method for providing early or prolonged enteral nutrition, especially for those developing surgical complications [36,37]. However, both NGT and NJT feeding are viable alternatives, and there is an ongoing debate about whether a feeding jejunostomy tube is always necessary at the time of esophagectomy [38].

Regarding PROs, our review found no statistically significant differences in the global health status/QoL score or any functional or symptom scales when comparing jejunostomy to no intervention, except for lower emotional functioning reported in one of the two studies reviewed. While the non-significant findings could suggest equivalent outcomes between the groups, it is also possible that the studies lacked sufficient statistical power to detect meaningful differences, especially given the small sample sizes. Similarly, no significant differences were found when comparing jejunostomy to NJT. However, comparisons between jejunostomy and NGT indicated that jejunostomy resulted in higher values of global health status and functioning (physical, role, and social) and lower levels of symptoms (fatigue, nausea/vomiting, pain, and appetite loss). Although these results are based on a high-quality RCT, with no significant baseline differences between groups, more studies are needed to confirm these results and better understand their clinical relevance.

### 4.2. Esophageal Stents vs. Alternatives

Most esophageal cancer patients present with locally advanced or metastatic disease at presentation [39], which is often associated with a significant symptom burden, mainly dysphagia [11]. Among the available options for palliative care in these patients, esophageal stents are frequently used to manage dysphagia or malignant fistula [10,12]. Four of the studies included in this systematic review, all of them classified as high-quality studies, evaluated the impact of esophageal stents on the QoL [19,23,28,29].

The results of studies comparing SEMSs with no specific intervention are inconsistent [19,23,28,29]. Several factors might, at least in part, explain these inconsistencies. Firstly, studies with smaller sample sizes may have lacked sufficient statistical power to detect meaningful differences. Secondly, there were differences in the QoL assessment methods, with three studies using the EORTC QLQ-C30 and one using the Spitzer QL Index, which may have introduced variability in how the QoL was captured. Third, the timing of evaluation also varied, with assessments conducted at twelve months, six weeks, and two weeks post-treatment in different studies, while another study reported mean differences between two time points, further complicating comparisons. Finally, the differences in control groups (patients receiving single-dose brachytherapy in one study versus no intervention in three studies) and the absence of control for confounding variables in all studies may also have contributed to these inconsistences.

Only one study compares SEMSs with laparoscopic gastrostomy [23] and another with NGT [28]. Therefore, caution is needed in drawing conclusions due to limited evidence.

Regarding pain, evidence from one study shows that a significantly higher proportion of patients experienced local severe pain following SEMS insertion compared to those undergoing RIG [26]. However, the authors did not specify the timing of pain evaluation, and therefore, this may reflect more acute pain related with the procedure rather than long-term outcomes. When comparing patients submitted to SEMSs with patients who received no specific intervention (but underwent radiotherapy or brachytherapy), the results were discrepant [19,25].

### 4.3. Selection Bias

No formal assessment of selection bias could be conducted due to the heterogeneity of methods used to present data. However, we cannot exclude the possibility that our strategy for selecting studies may have resulted in some eligible studies being omitted. However, efforts have been made to decrease this possibility, such as using the backward citation tracking of the included studies and the employment of software to extract data from figures (otherwise, these studies would have been excluded).

### 4.4. Limitations of Current Research and Future Approaches

Despite the recognized importance of PROs as important prognostic variables in esophageal cancer patients [40], there is still a limited number of studies evaluating these outcomes in patients submitted to different techniques for nutritional support. For a better understanding of the impact of different techniques on PROs, it is paramount to collect more robust longitudinal data and provide a more comprehensive report of the results. For instance, among the studies included, only three were RCTs. Also, among the nine studies evaluating the QoL, only three provide data on the variation in scores between the baseline and follow-up. Although four out of nine non-randomized studies conducted a baseline evaluation before patients underwent various techniques [17,22,27,28], they often only reported mean values at baseline (before any intervention) and follow-up [22] across groups. This approach, rather than presenting the mean variation among groups over time, makes it difficult to evaluate all the results collectively. Also, the differences in the QoL and pain measurement tools may affect comparability between studies. However, since most studies used the QLQ-C30 for QoL assessment, this consistency likely minimized the impact of measurement variation on the results related to the QoL.

Another important issue is the variability in methodological quality across the included studies, which may have compromised their comparability and led to the potential misestimation of associations, particularly in non-randomized studies, due to the lack of control for confounding variables. As previously noted, only three of the eight non-randomized studies in this systematic review provided adjusted estimates. Among the many potential confounding variables, cancer stage and tumor location appear to be particularly important. In fact, treatment decisions depend on cancer stage and tumor location [41,42,43], and these factors may both influence the technique used for nutritional support [10,11,12] and PROs [44]. Additionally, socioeconomic background [45,46,47] and the presence of other heath conditions [46] should also be considered as potential confounders in future studies evaluating the impact of techniques used for the nutritional support of esophageal cancer patients and PROs.

In addition to the limited number of studies, future research needs to improve the evaluation of outcomes. In fact, several studies were excluded because they did not use standardized measures for data collection. For instance, some studies reported dysphagia or pain severity based on retrospective data from health records without specifying whether standardized patient-reported measures were used. Previous studies have demonstrated that, in addition to increasing patients’ engagement with the clinical team and the care provided, the use of PROs improves patients’ QoL and prognosis [48,49], and therefore, their use needs to be encouraged both in research and in routine clinical practice.

## 5. Conclusions

This review underscores the importance of considering PRO measures when evaluating esophageal cancer patients, namely regarding nutritional support techniques. However, further robust research is needed to fully understand the optimal approaches for improving patients’ wellbeing. Until more comprehensive data are available, clinical decision-making should be individualized, considering the patient’s condition and existing guidelines.

## Figures and Tables

**Table 1:** Description of the methods used for evaluation of patient-reported outcomes.

Studies Included in the Systematic Review (1st Author, Year of Publication)	PROs Reported and Instrument Used for Their Evaluation							
	Overall Quality of Life					Specific Symptoms		
						Pain		Depression
	EORTC QLQ-C30 [30]	EORTC QLQ-OES18 [31]	EORTC QLQ-OES23 [32]	Euroqol (EQ)-5D [33]	Spitzer QL Index [34]	Visual Analog Scale	Likert Scale	Depression Module of the Patient Health Questionnaire (PHQ-9) [35]
Dong, 2023 [22]	✓							
Wang, 2021 [26]						✓		
Martin, 2020 [25]							✓	
Tao, 2020 [18]	✓							
Klevebro, 2018 [24]	✓	✓						
Wu, 2018 [27]	✓					✓		
Yu, 2018 [28]	✓	✓						✓
Scarpa, 2014 [17]	✓	✓						
Hu, 2009 [23]	✓	✓						
Shenfine, 2009 [29]	^a^			✓	✓			
Homs, 2004 [19]	✓		✓	✓		✓		

^a^ The authors only reported data from the pain symptom score of the EORTC QLQ-C30, and therefore, this study was not considered for Figure 1 and Figure 2.

**Table 2:** Assessment of methodological quality of studies included in the systematic review, using the Mixed Methods Appraisal Tool, version 2018.

Category of Study Designs	Methodological Quality Criteria	Studies Included in the Systematic Review										
		Tao, 2020 [18]	Shenfine, 2009 [29]	Homs, 2004 [19]	Dong, 2023 [22]	Wang, 2021 [26]	Martin, 2020 [25]	Klevebro, 2018 [24]	Wu, 2018 [27]	Yu, 2018 [28]	Scarpa, 2014 [17]	Hu, 2009 [23]
Quantitative randomized controlled trials	Is randomization appropriately performed?	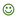	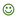	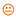	--	--	--	--	--	--	--	--
	Are the groups comparable at baseline?	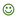	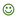	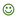	--	--	--	--	--	--	--	--
	Are there complete outcome data?	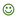	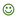	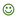	--	--	--	--	--	--	--	--
	Are the outcome assessors blinded to the intervention provided?	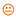	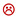	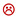	--	--	--	--	--	--	--	--
	Did the participants adhere to the assigned intervention?	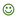	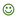	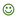	--	--	--	--	--	--	--	--
Quantitative non-randomized	Are the participants representative of the target population?	--	--	--	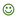	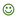	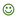	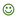	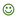	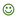	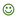	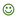
	Are measurements appropriate regarding both the outcome and intervention (or exposure)?	--	--	--	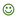	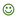	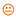	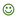	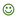	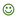	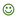	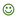
	Are there complete outcome data?	--	--	--	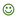	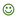	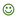	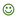	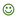	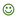	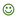	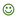
	Are the confounders accounted for in the design and analysis?	--	--	--	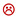	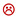	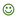	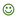	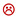	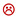	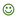	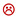
	During the study period, is the intervention administered (or exposure occurred) as intended?	--	--	--	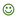	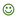	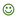	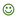	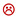	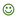	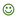	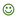

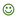
 yes; 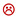 no; 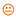 the article does not report appropriate information to answer “Yes” or “No” or unclear information related to the criterion was reported.

## Data Availability

No new data were created or analyzed in this study. Data sharing is not applicable to this article.

## Appendix A

**Table A1:** Detailed description of the studies evaluating the impact of surgical, endoscopic, or radiological techniques for nutritional support in esophageal cancer patients on patient-reported outcomes.

1st Author, Year of Publication (REF)	Study Description	Subjects’ Characteristics	Technique Evaluated	PRO Evaluated (PRO Measurement)	Timing of Assessment and Results Presented	Control of Confounding
Dong, 2023 [22]	Country: China. Study design: Prospective cohort study. Aim: To analyze the differences in the nutritional status, chemoradiotherapy-related adverse events, and QoL of patients with esophageal cancer receiving nutrition through oral intake, PEG, and NGT approaches during concurrent chemoradiotherapy. Study population: Patients with esophageal squamous cell carcinoma (18–75 years) who were scheduled to receive concurrent chemoradiotherapy. Sample size: N = 141; “PEG group”: N = 74; “NGT group”: N = 29; “Control group (oral intake)”: N = 38.	Age (number [%]): <60 years: 36 (48.6%) vs. 11 (37.9%) vs. 13 (34.2%); ≥60 years: 38 (51.4%) vs. 18 (62.1%) vs. 25 (65.8%) in the PEG, NGT, and control groups, respectively. Cancer stage (number [%]): stage II-III: 37 (50.0%) vs. 10 (34.5%) vs. 26 (68.4%); stage IV: 37 (0.0%) vs. 19 (65.5%) vs. 12 (31.6%) in the PEG, NGT, and control groups, respectively. Type of carcinoma: squamous cell carcinoma.	PEG vs. NGT vs. none	QoL (EORTC QLQ-C30)	Mean (SD) in scores before and after concurrent chemoradiotherapy.	--
Wang, 2021 [26]	Country: China. Study design: Retrospective cohort study. Aim: To compare the changes in nutritional status, the safety of the procedures, and the overall survival of patients with esophageal cancer and dysphagia who underwent stent insertion or percutaneous gastrostomy. Study population: Patients with dysphagia who underwent gastrostomy or stent insertion. Sample size: N = 113; “SEMS group”: N = 47; “RIG group”: N = 66.	Age (median [IQR]): 76.0 (9.0) vs. 75.0 (6.0) years in the SEMS and RIG groups, respectively. Cancer stage (number [%]): stage II/III: 22 (31.9%) vs. 20 (30.3%); stage IV: 25 (68.1%) vs. 46 (69.7%) in the SEMS and RIG groups, respectively. Type of carcinoma (number [%]): squamous cell carcinoma: 38 (80.9%) vs. 60 (90.9%); adenocarcinoma: 9 (19.1%) vs. 6 (9.1%) in the SEMS and RIG groups, respectively.	SEMS vs. RIG	Pain (Visual Analog Scale)	Number (%) of patients with local severe pain (score of 7 or greater).	--
Martin, 2020 [25]	Country: United States of America. Study design: Retrospective cohort study. Aim: To assess the efficacy and complications of palliative external-beam radiotherapy compared with esophageal stent placement among a large, multi-institutional cohort of veterans diagnosed with metastatic esophageal cancer. Study population: Veterans with esophageal cancer stage IV, diagnosed between 2000 and 2015, treated with palliative external-beam radiotherapy and/or esophageal stenting. Sample size: N = 766; “Stent group”: N = 31%; “Control group (Radiotherapy)”: N = 41%.	Age: -- Cancer stage: stage IV. Type of carcinoma: --	Stent ^1^ vs. none	Pain [Likert scale (0–10)]	Estimate from linear mixed effects model.	Charlson comorbidity index score, income, population density, and tumor location.
Tao, 2020 [18]	Country: China. Study design: Randomized controlled trial. Aim: To assess two common enteral nutrition methods after minimally invasive McKeown esophagectomy. Study population: Patients who underwent minimally invasive McKeown esophagectomy. Sample size: N = 120; “Jejunostomy group”: N = 58; “NGT group”: N = 62.	Age (mean [range]): 65 (41–81) vs. 64 (39–82) years in the jejunostomy and NGT groups, respectively. Cancer stage (number): stage I: 12 (20.7) vs. 19 (30.6); stage II: 21 (36.2) vs. 23 (37.1); stage III: 16 (27.6) vs. 16 (25.8); stage IV: 9 (12.6) vs. 4 (6.5) in the jejunostomy and NGT groups, respectively. Type of carcinoma: squamous cell carcinoma.	Surgical jejunostomy vs. NGT	QoL (EORTC QLQ-C30)	Mean (SE) scores preoperatively and one week and one month after surgery.	--
Klevebro, 2018 [24]	Country: Sweden. Study design: Prospective cohort study. Aim: To assess the influence of jejunostomy on postoperative outcomes other than weight development in patients who underwent esophagectomy for esophageal cancer. Study population: Patients operated on for esophageal or gastroesophageal junctional cancer. Sample size: N = 397; “Jejunostomy group”: N = 178; “Control group”: N = 219.	Age (number [%]): <60 years: 41 (23%) vs. 58 (26%); 60–74 years: 102 (57%) vs. 128 (58%); >74 years: 35 (20%) vs. 33 (15%) in the jejunostomy and control groups, respectively. Cancer stage (number [%]): stage 0-I: 39 (22%) vs. 44 (20%); stage II: 51 (29%) vs. 69 (32%); stage III: 72 (40%) vs. 89 (41%); stage IV: 16 (9%) vs. 17 (8%) in the jejunostomy and control groups, respectively. Type of carcinoma (number [%]): squamous cell carcinoma: 53 (30%) vs. 44 (20%); adenocarcinoma: 125 (70%) vs. 175 (80%) in the jejunostomy and control groups, respectively.	Surgical jejunostomy vs. none	QoL (EORTC QLQ-C30 and EORTC QLQ-OES 18)	Adjusted mean scores (95% CI) six months after esophagectomy.	Age, sex, histological tumor type, tumor stage, Charlson comorbidity index, surgical technique, neoadjuvant therapy, baseline body mass index, and weight loss at baseline.
Wu, 2018 [27]	Country: China. Study design: Prospective cohort study. Aim: To investigate the effect of 3 months home enteral nutrition on health-related QoL and nutritional status of esophageal cancer patients who were preoperatively malnourished. Study population: Patients diagnosed with esophageal and esophagogastric junction cancer and suitable for potentially curative resection with intrathoracic anastomosis. Sample size: N = 142; “Jejunostomy group”: N = 67; “NJT group”: N = 75.	Age (median [range]): 62 (45–80) vs. 61 (43–80) years in the jejunostomy and NJT groups, respectively. Cancer stage (number [%]): stage 0/I: 13 (19.4%) vs. 10 (13.3%); stage II: 27 (40.3%) vs. 36 (48.0%); stage III: 27 (40.3%) vs. 29 (38.6%) in the jejunostomy and NJT groups, respectively. Type of carcinoma (number [%]): squamous cell carcinoma: 61 (91.0%) vs. 71 (94.7%); adenocarcinoma: 6 (9.0%) vs. 4 (5.3%) in the jejunostomy and NJT groups, respectively.	Surgical jejunostomy vs. NJT	QoL (EORTC QLQ-C30)	Mean (SD) scores before surgery and two weeks and three months after surgery.	--
				Pain (Visual Analog Scale)	Median (range) scores within 72 h post-surgery.	--
Yu, 2018 [28]	Country: Taiwan. Study design: Non-randomized controlled trial. Aim: To evaluate esophageal squamous cell carcinoma patients who received neoadjuvant or definite chemoradiation radiotherapy to compare the efficacy, safety, and QoL among patients using different methods to maintain enteral nutrition. Study population: Incident esophageal squamous cell carcinoma patients who received neoadjuvant or definite chemoradiation radiotherapy. Sample size: N = 81; “SEMS group”: N = 7; “Jejunostomy/gastrostomy group”: N = 26; “NGT group”: N = 19; “Control group (oral intake)”: N = 29.	Age (mean [SD]): 57.2 (13.7) vs. 57.8 (7.8) vs. 53.6 (6.7) vs. 57.8 (9.8) years in the SEMS, jejunostomy/gastrostomy, NGT, and control groups, respectively. Cancer stage (number [%]): stage I/II: 0 (0%) vs. 3 (11.5%) vs. 1(5.3%) vs. 3 (10.3); stage III: 7 (100.0%) vs. 19 (73.1%) vs. 16 (84.2) vs. 19 (65.5%); stage IV: 0 (0.0%), 4 (15.4%), 2 (10.5%), 7 (24.1%) in the SEMS, jejunostomy/gastrostomy, NGT, and control groups, respectively. Type of carcinoma: squamous cell carcinoma.	SEMS vs. surgical jejunostomy/gastrostomy vs. NGT vs. none	QoL (EORTC QLQ-C30 and EORTC QLQ-OES18)	Mean difference (between 4 and 5 weeks after completion of CRT and at cancer diagnosis) in the scores.	--
				Depression (Patient Health Questionnaire)	Mean difference (between 4 and 5 weeks after completion of CRT and at cancer diagnosis) in the scores.	--
Scarpa, 2014 [17]	Country: Italy. Study design: Retrospective cohort study. Aim: To evaluate the impact of jejunostomy during esophagectomy for cancer on postoperative health-related QoL. Study population: Patients who underwent esophagectomy for cancer. Sample size: N = 109; “Jejunostomy group”: N = 40; “Non-jejunostomy group”: N = 69.	Age (median [IQR]): 64 (58–71) vs. 59 (51–67) in the jejunostomy and non-jejunostomy groups, respectively. Cancer stage (number [%]): stage 0: 7 (17.5%) vs. 18 (26.1%); stage I-II: 12 (30.0%) vs. 31 (44.9%); stage III-IV: 21 (54.5%) vs. 20 (29.9%) in the jejunostomy and non-jejunostomy groups, respectively. Type of carcinoma (number [%]): squamous cell carcinoma: 21 (53.8%) vs. 12 (17.4%); adenocarcinoma: 18 (46.2%) vs. 57 (52.6%) in the jejunostomy and non-jejunostomy groups, respectively.	Surgical jejunostomy vs. none	QoL (EORTC QLQ-C30 and EORTC QLQ-OES 18)	Mean (SD) scores at admission and after surgery and mean (SD) score differences between scores at admission and after surgery.	General linear models adjusted for age, tumor site, and pathologic stage ^2^.
Shenfine, 2009 [29]	Country: United Kingdom. Study design: Randomized controlled trial. Aim: To compare the clinical effectiveness and cost-effectiveness of SEMSs with other palliative therapies. Study population: Patients with dysphagia due to esophageal cancer. Sample size: N = 101; “SEMS group”: N = 53 ^3^; “Control group (non-SEMS group)”: N = 48.	Age (mean [SD]): 74.6 (11.4) vs. 76.9 (9.0) in the SEMS and control groups, respectively. Cancer stage: -- Type of carcinoma: squamous cell carcinoma: 15 (28.3%) vs. 16 (32.0%); adenocarcinoma: 32 (60.4%) vs. 31 (62.0%) in the SEMS and control groups, respectively.	SEMS vs. none	QoL (Spitzer QL Index and Euroqol (EQ)-5D)	Mean (SD) scores at admission and six weeks after.	--
Hu, 2009 [23]	Country: China. Study design: Nan-randomized controlled trial. Aim: To compare the survival time and QoL of patients who have received different treatments for tracheoesophageal/bronchoesophageal fistula. Study population: Patients diagnosed with tracheoesophageal/ bronchoesophageal fistula secondary to esophageal squamous. Sample size: N = 27; “Gastrostomy group”: N = 7; “SEMS group”: N = 13; “Control group (conservative therapy)”: N = 7.	Age (mean [SD]): 57.33 (8.60) vs. 56.83 (7.72) vs. 59.56 (4.77) years in the gastrostomy, SEMS, and control groups, respectively. Cancer stage: -- Type of carcinoma: squamous cell carcinoma.	Surgical gastrostomy vs. SEMS vs. none	QoL (EORTC QLQ-C30 and EORTC QLQ-OES 18)	Median (IQR) scores two weeks after gastrostomy or stent insertion (two weeks after admission for the control group).	--
Homs, 2004 [19]	Country: The Netherlands. Study design: Randomized controlled trial. Aim: To compare metal stent placement and single-dose brachytherapy with respect to generic and disease-specific health-related QoL. Study population: Patients with inoperable cancer of the esophagus or esophagogastric junction due to metastatic disease and/or a poor medical condition with progressive dysphagia. Sample size: N = 209; “SEMS group”: N = 108; “Control group (Single dose brachytherapy)”: N = 101.	Age (mean [SD]): 69 (11) vs. 69 (13) in the SEMS and control groups, respectively. Cancer stage: -- Type of carcinoma (number [%]): squamous cell carcinoma: 29 (27%) vs. 29 (29%); adenocarcinoma: 75 (69%) vs. 69 (68%); other: 4 (4%) vs. 3 (3%) in the SEMS and control groups, respectively.	SEMS vs. none	QoL (EORTC QLQ-C30, EORTC QLQ OES-23, and Euroqol (EQ)-5D)	Mean (95%CI) scores before treatment and 12 months after treatment.	--
				Pain (Visual Analog Scale)	Mean (95%CI) scores before treatment and 12 months after treatment.	--

NGT, nasogastric tube; NJT, nasojejunal tube; PEG, percutaneous endoscopic gastrostomy; QoL, quality of life; RIG, radiologically inserted gastrostomy; SD, standard deviation; SE, standard error; SEMS, self-expandable metal stent. ^1^ The type of stent was not described by the authors. ^2^ The control of confounding was only performed by the authors for the mean (SD) score differences between scores at admission and after surgery. ^3^ This study depicted data from patients submitted to rigid stent, non-stent, SEMS 18 mm, and SEMS 24 mm. Data were retrieved for the control group (non-stent) and the SEMS group with the highest sample size (SEMS 24 mm).
